# Editorial: Diseases with immune dysregulation in Africa

**DOI:** 10.3389/fimmu.2026.1941384

**Published:** 2026-08-04

**Authors:** Lydia Lamara Mahammed, Merzak Gharnaout, Najla Mekki, Reda Djidjik

**Affiliations:** 1 Department of Medical Immunology, Beni-Messous Teaching Hospital, Algiers, Algeria; 2 Faculty of Pharmacy, University of Health Sciences, Algiers, Algeria; 3 Department of Pulmonology and Allergy, Beni-Messous Teaching Hospital, Algiers, Algeria; 4 Faculty of Medicine, University of Health Sciences, Algiers, Algeria; 5 Department of Immunology, Pasteur Institute of Tunis, Tunis, Tunisia; 6 Faculty of Medicine of Tunis, Tunis El-Manar University, Tunis, Tunisia

**Keywords:** allergy, autoimmune diseases, dysregulation, inborn errors of immunity, North- Africa

## Introduction

Immune dysregulation encompasses a broad spectrum of disorders, including inborn errors of immunity (IEIs), autoimmune and autoinflammatory diseases, allergic disorders, and cancers. Although traditionally considered distinct entities, these disorders are increasingly recognized as interconnected manifestations of impaired immune homeostasis resulting from diverse alterations affecting immune development, regulation, and function. Advances in molecular and cellular technologies have profoundly reshaped our understanding of these diseases, shifting clinical immunology toward an integrated, mechanism-based discipline.

This evolving understanding is particularly relevant in Africa. While infectious diseases continue to represent an important public health challenge, immune-mediated disorders are becoming increasingly recognized across the continent. Nevertheless, evidence from African populations remains limited, resulting in substantial gaps in epidemiological data, genetic characterization, and disease-specific diagnostic and management strategies. This knowledge gap is especially important in North Africa, where high rates of consanguinity increase the burden of inherited disorders and underscore the need for earlier diagnosis and improved access to specialized care.

To address these unmet needs, this Research Topic brings together 11 contributions, comprising six original research articles (Tahiat et al., Ben Khaled et al., Ben Nejma et al., Hadjout et al., Meddeb et al. and Tajouri et al.), four case reports (Kattra et al., Yaakoubi et al., Belaid et al. and Abida et al.) and one review (Djidjik et al.). Together, these contributions illustrate recent advances in the understanding of immune dysregulation through investigations originating from North Africa that span the spectrum of immune dysregulation diseases, from monogenic disorders to more complex multifactorial conditions.

## Inborn errors of immunity: natural models of immune dysregulation

Several contributions in this Research Topic substantially expand current knowledge of IEIs in African populations. Among the original contributions, Tahiat et al. provide one of the largest African cohorts investigating immune dysregulation in IEIs. In a cohort of 825 Algerian patients, autoimmune and/or autoinflammatory manifestations affected 26.3% of individuals and represented the initial clinical presentation in nearly half of the affected patients, emphasizing that immune dysregulation is often an early hallmark rather than a late complication of these disorders.

Expanding the molecular landscape of IEIs, Belaid et al. and Kattra et al. describe two novel pathogenic variants. Belaid et al. identified a novel homozygous frameshift mutation causing CD19 complex deficiency in an Algerian family, providing additional insights into the critical role of CD19 in humoral immune responses. Similarly, Kattra et al. reported the first Moroccan family with a novel pathogenic *CIITA* mutation responsible for MHC class II deficiency, thereby expanding the regional mutational spectrum of this disorder.

Beyond the discovery of novel disease-causing variants, advances in genomic technologies are also revealing the increasing complexity of the genetic architecture underlying IEIs. Yaakoubi et al. report a unique dual molecular diagnosis combining a pathogenic *STAT1* gain-of-function mutation with a regulatory *STAT3* variant, demonstrating how multiple genetic alterations, including variants located outside coding regions, may interact to generate atypical and overlapping clinical phenotypes.

Complementing these findings, Tajouri et al. identified the recurrent *CFI* p.Ile357Met pathogenic variant as a hotspot mutation in Tunisian patients with atypical hemolytic uremic syndrome, highlighting the importance of recognizing population-specific variants and potential founder effects to facilitate targeted molecular screening and earlier diagnosis.

Collectively, these original studies and case reports illustrate the progressive implementation of genomic technologies in routine clinical practice across North Africa. Beyond establishing definitive molecular diagnoses, these approaches are expanding the mutational spectrum of IEIs, refining genotype–phenotype correlations, improving genetic counseling, and increasingly guiding therapeutic decision-making.

## Autoimmune diseases: refining diagnosis and improving patient stratification

Autoimmune diseases represent one of the most common manifestations of immune dysregulation and remain a major clinical challenge because of their marked heterogeneity. In this context, Ben Khaled et al. identified predictors of disease severity and poor treatment response in pediatric Evans syndrome. Their study demonstrated that severe disease was associated with younger age and severe anemia at presentation, whereas hepatomegaly and abnormal IgM levels predicted the need for second-line therapy. In parallel, Meddeb et al. show that anti-neutrophil cytoplasmic antibodies, particularly anti-lactoferrin, are associated with higher disease activity and more severe renal involvement in Tunisian patients with systemic lupus erythematosus, supporting the development of biomarkers for disease stratification.

Advances in laboratory diagnostics are illustrated by Hadjout et al., who evaluate solid-phase automated assays for antinuclear antibody (ANA) detection, demonstrating how modern technologies may complement conventional indirect immunofluorescence and improve the diagnosis of ANA-associated rheumatic diseases.

Finally, broaden the spectrum of immune dysregulation disorders reported in North Africa by describing the first regional case of anti-HMGCR immune-mediated necrotizing myopathy. This Tunisian case illustrates the diagnostic value of disease-specific autoantibody testing, and highlights the therapeutic challenges associated with treatment-resistant immune-mediated disorders.

## Allergic diseases: advancing molecular allergology

Although represented by a single original contribution, allergic diseases represent an essential component of the immune dysregulation spectrum. Ben Nejma et al. provide the first comprehensive molecular characterization of peach allergy in Tunisia and North Africa, demonstrating that regional sensitization patterns largely reflect the Mediterranean molecular profile, with predominant Pru p 3 sensitization and significant clinical severity. Beyond generating valuable epidemiological data, this study illustrates the ongoing transition from extract-based diagnosis toward molecular allergology across North Africa, enabling more accurate diagnosis, improved risk assessment, and personalized patient management.

## The JAK–STAT pathway: a paradigm of immune homeostasis

One contribution in this Research Topic highlights a fundamental molecular pathway that bridges the diverse spectrum of immune dysregulation disorders. In their comprehensive review, Djidjik et al. describe the JAK–STAT signaling pathway as a central regulator of immune homeostasis, illustrating how disturbances of a single signaling cascade may give rise to remarkably diverse clinical phenotypes, including immunodeficiency, autoimmunity, autoinflammation, allergic manifestations, and hematologic malignancies. This review provides the mechanistic framework that connects many of the clinical observations reported throughout this Research Topic and highlights how advances in the understanding of disease mechanisms are rapidly translating into targeted therapeutic strategies and precision medicine.

## Looking ahead

The studies brought together in this Research Topic collectively demonstrate that immunological research in Africa is entering a new era. Studies conducted in African cohorts are not only validating existing knowledge but also expanding it through the identification of novel pathogenic variants, the elucidation of distinctive genotype–phenotype relationships and the characterization of population-specific biomarker profiles. Moreover, these studies address important regional healthcare priorities while contributing to improved diagnosis and patient management. Continued investment in collaborative research, advanced immunological diagnostics, and research infrastructure across the continent will further strengthen Africa’s contribution to unraveling disease mechanisms and foster internationally relevant discoveries.

